# Neurovascular dysfunction in vascular dementia, Alzheimer’s and atherosclerosis

**DOI:** 10.1186/s12868-018-0465-5

**Published:** 2018-10-17

**Authors:** Osman Shabir, Jason Berwick, Sheila E. Francis

**Affiliations:** 1 The Neurovascular and Neuroimaging Research Group, Alfred Denny Building, The University of Sheffield, Western Bank, Sheffield, S10 2TN UK; 2 Department of Infection, Immunity and Cardiovascular Disease, The University of Sheffield, Medical School, Beech Hill Road, Sheffield, S10 2RX UK

**Keywords:** Neurovascular coupling, Atherosclerosis, Dementia, Mouse, Neuroimaging, Disease modelling, Alzheimer’s, Vascular dementia

## Abstract

Efficient blood supply to the brain is of paramount importance to its normal functioning and improper blood flow can result in potentially devastating neurological consequences. Cerebral blood flow in response to neural activity is intrinsically regulated by a complex interplay between various cell types within the brain in a relationship termed neurovascular coupling. The breakdown of neurovascular coupling is evident across a wide variety of both neurological and psychiatric disorders including Alzheimer’s disease. Atherosclerosis is a chronic syndrome affecting the integrity and function of major blood vessels including those that supply the brain, and it is therefore hypothesised that atherosclerosis impairs cerebral blood flow and neurovascular coupling leading to cerebrovascular dysfunction. This review will discuss the mechanisms of neurovascular coupling in health and disease and how atherosclerosis can potentially cause cerebrovascular dysfunction that may lead to cognitive decline as well as stroke. Understanding the mechanisms of neurovascular coupling in health and disease may enable us to develop potential therapies to prevent the breakdown of neurovascular coupling in the treatment of vascular brain diseases including vascular dementia, Alzheimer’s disease and stroke.

## Background

The human brain accounts for just 2% of total body weight, yet receives between 15 and 20% of total cardiac output [1]. It is therefore evident that the brain requires an efficient and adequate blood supply to support the metabolic demands it exerts. Unlike the majority of organs and tissues, the brain has a tightly regulated blood brain barrier (BBB), which prevents leakage of blood into the parenchyma and protects the brain from toxic agents and infection. Neurons, therefore, are not in direct association with the blood, but are functionally and structurally connected to a network of cell types within a structure called the neurovascular unit (NVU) [2]. The NVU facilitates cerebral blood flow (CBF) changes in response to the metabolic activity of neurons in a relationship termed neurovascular coupling [3]. Neurovascular coupling ensures that the brain has a proportionally matched CBF in response to neural activity, however dysfunction of neurovascular coupling, either caused by pathology or ageing itself, can cause further cerebral pathologies and neurological disorders. This review will focus on the neurophysiology of neurovascular coupling, how it is impaired in certain neurological conditions, how to study it and will discuss how cardiovascular disease may affect the regulation of CBF and neurovascular coupling.

## Neurovascular coupling

### The neurovascular unit and the blood brain barrier

**Fig. 1:**
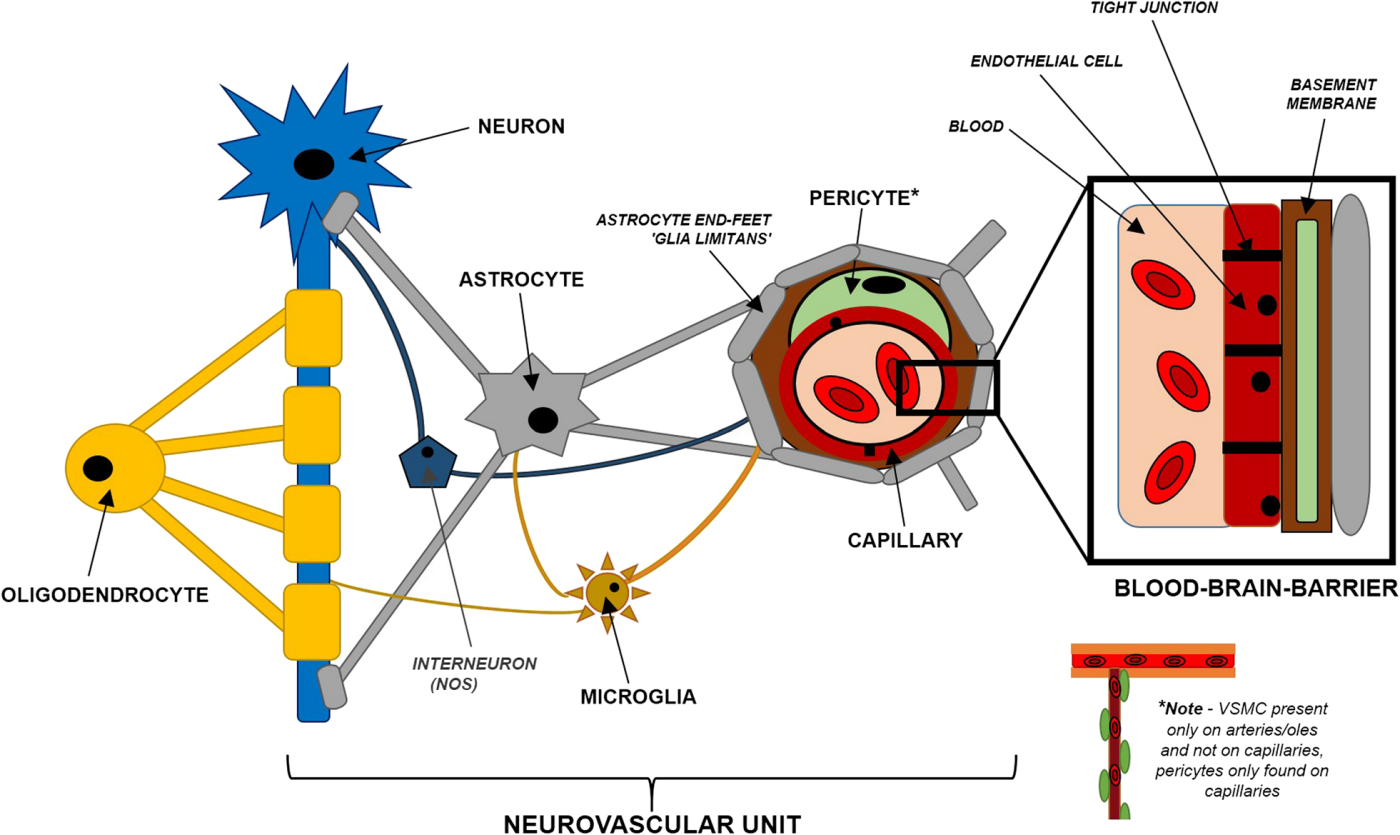
The neurovascular unit and the BBB at a capillary. Cerebral arteries have extensive vascular smooth muscle cell (VSMC) coverage regulating vasoconstriction. Capillaries on the other hand lack vascular smooth muscle cells, and are instead covered by small pericytes which maintain the integrity of the BBB, as well as regulate their diameter. Cerebral vessels receive inputs from astrocytes by their end-feet to regulate arteriolar diameter, as well as inputs from various interneuron groups

The NVU consists of neurons, astrocytes, microglia, interneurons, pericytes, vascular smooth muscle cells (VSMCs) and endothelial cells [2]. It includes the different cell types involved in the structural and functional connection between neurons and blood vessels in the brain, including cells involved in the formation of the BBB, *refer to* Fig. 1. The BBB is formed due to the specialised functions of tight-junctions of endothelial cells in cerebral vasculature, astrocytes, with some evidence suggesting a crucial role for pericytes [4]. The BBB is a specialised structure in the cerebral vasculature, and is not seen in many other organs. It serves to limit the entry of pathogens, toxic agents and blood cells into the parenchyma [5], protecting the brain from infection, whilst allowing controlled transport of nutrients back and forth from the brain. However, there are natural pathogens that can penetrate the BBB including group B *streptococci*, which can cause meningitis [6]. Generic or specialised dysfunction of the NVU is associated with an increasing list of pathologies, including stroke, Parkinson’s disease, Alzheimer’s disease, multiple sclerosis, amyotrophic lateral sclerosis and vascular dementia [2]. Specific pathologies as a result of NVU and BBB dysfunction will be discussed later.

### Neurophysiology of neurovascular coupling

**Fig. 2:**
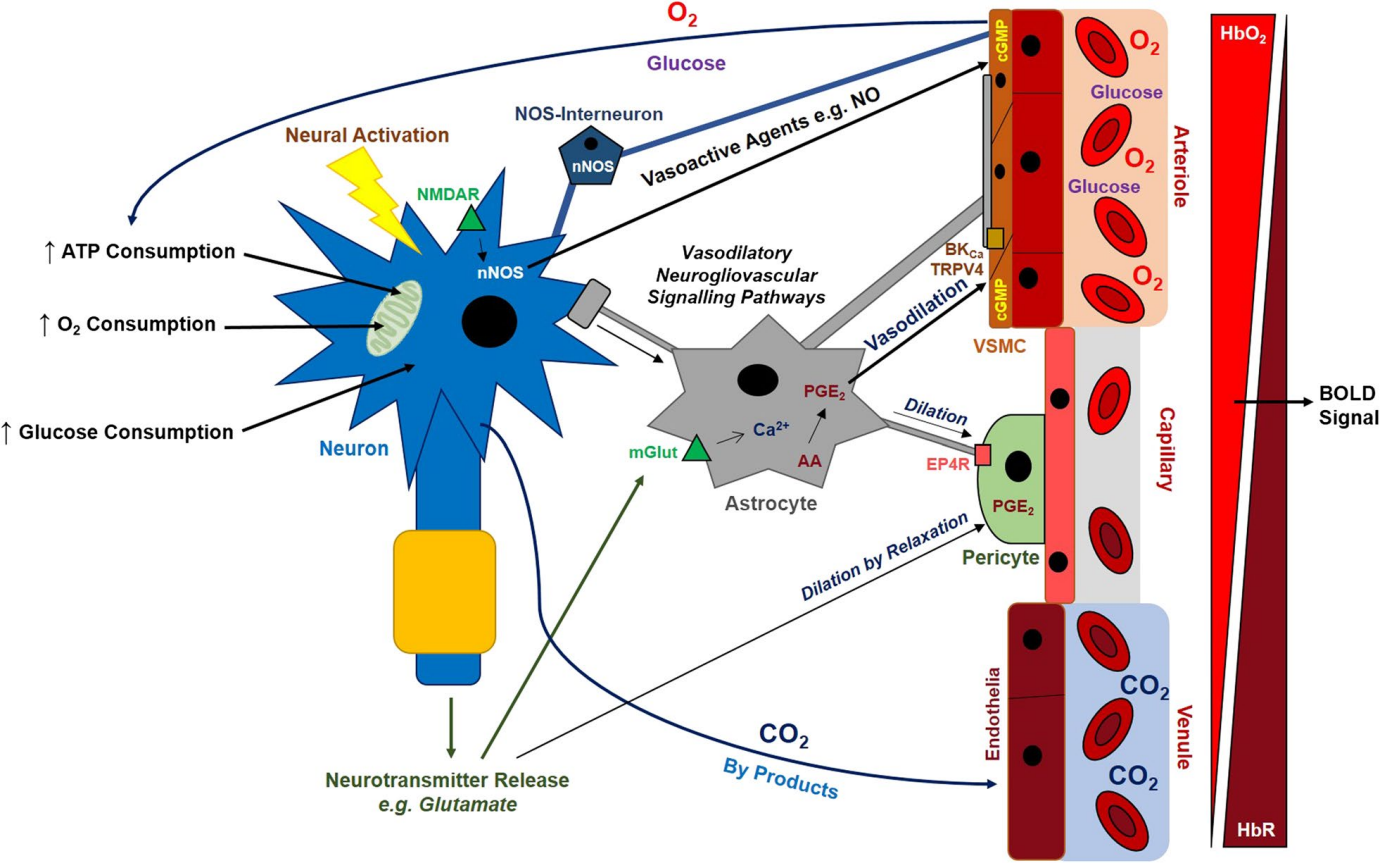
Neurophysiology of neurovascular coupling overview. Neural activity is metabolically expensive demanding a high consumption of glucose and oxygen (from arterial blood). Simultaneously, CO_2_ and other by products are also produced, which need to be removed by diffusion into venous blood in order to prevent hypercapnia and acidosis. In order to achieve this, neurons regulate blood flow via neurovascular coupling. Vasoactive agents such as NO directly cause vasodilation of arterial smooth muscle cells. Significant neurogliovascular signalling involving glutamate and calcium signalling causes vasodilation via channels on VSMCs e.g. BK_Ca_, TRPV4, to stimulate cGMP [105]. There are indeed multiple and complex signalling pathways (including ATP and VIP signalling) also involved in neurovascular coupling. In addition to vasodilator signals, vasoconstrictive signals are also produced, namely 20-HETE, however this is thought to be pathological and as a result of ageing [106]. Pericytes regulate capillary diameter by responding to glutamate release, in addition to PGE_2_ produced by astrocytes

The NVU facilitates haemodynamic changes (alteration in CBF) in response to neural activity. This relationship is termed neurovascular coupling or functional hyperaemia, and is essential for normal metabolic functioning of neurons and the brain as a whole [3], Fig. 2. Neurovascular coupling is thought to be governed by direct neural and endothelial interactions, or through complex neurogliovascular signalling pathways (of which some are highlighted in Fig. 2). Neural activation causes neurotransmitter release from synaptic terminals (e.g. glutamate), which bind to *N*-methyl-d-aspartate receptors (NMDARs) or metabotropic glutamate receptors (mGlutRs) on nNOS-expressing interneurons initiates the synthesis of nitric oxide (NO) [7]. NO can directly cause vasodilation in the endothelium by stimulating cGMP in VSMCs. NO also inhibits CYP4A, which is needed to produce 20-HETE; a prominent vasoconstrictor [8].

Most neurons do not directly innervate the overall vasculature; rather glial intermediaries make the majority of neural-mediated inputs (refer to Fig. 1). With respect to neurogliovascular signalling, the most important players are thought to be astrocytes whose terminal processes called vascular end-feet, wrap around blood vessels in the brain to contribute to BBB integrity, as well as facilitating neurovascular coupling. Calcium, sodium and potassium signalling via the astrocytic-endothelial axis within the NVU may promote a slow and prolonged vasodilation compared to neural outputs, however this is contested by Lauritzen’s group who suggest that astrocytes are involved in the initial vasodilation [9]. On the other hand, rapidly induced transient vasodilation caused by neural NO [10], ensures that CBF change is sufficient to match the demands of metabolic expenditure. Furthermore, it has been shown that a transient loss of oxygen and glucose (as in ischaemia) causes excitotoxicity in neurons leading to delayed neuronal death [11]. At the centre of this excitotoxicity is a delayed mitophagy response (loss of mitochondria) from astrocyte end-feet, leading to a toxic increase in intracellular calcium [Ca^2+^]_IC_ and glutamatergic activation [11], leading to neuronal death. Although functional neurovascular coupling is related to increased neural activity (i.e. stimulation of brain areas), the brain at rest also requires an efficient steady supply of blood flow. Astrocytes have been shown to influence arteriolar tone by a steady low-level efflux of prostaglandin-E2 (PGE_2_) as a result of basal [Ca^2+^]_IC_ fluctuations in astrocytic end-feet [12]. This basal tonic influence of arteriolar diameter is independent from stimulus-evoked neural activity-dependent haemodynamic changes, without affecting stimulus-evoked functional hyperaemia. It is therefore assumed that astrocyte dysfunction is key to the breakdown of neurovascular coupling and therefore many neurological conditions.

In addition to astrocytes, interneurons also play a role in neurovascular coupling. Interneurons in the cortex come in distinct varieties: VIP/ChAT, NOS/NPY, SOM [7] as well as paravalbumin-*GABAergic* interneurons [13]. Depending on the input these interneurons receive, they have different outputs with respect to vasoconstriction or vasodilation. For example, acetylcholine (ACh) binding to muscarinic receptors on NOS-interneurons causes the release of NO to facilitate vasodilation on nearby micro vessels, however a serotonergic (5-HT) input on the same interneurons can cause the release of NPY, which can facilitate vasoconstriction [7]. The role of interneurons in neurovascular coupling is still poorly understood and elucidating the mechanistic pathways involved is still to be fully investigated.

As arterioles turn into capillaries in the parenchyma, cerebral vessels no longer have a coverage of VSMCs and instead have a scattered covering of specialised contractile cells called pericytes [14]. Pericytes are an important component of the BBB and maintain its integrity by regulating adherens junction proteins on endothelial cells [15]. The exact function and role of pericytes in the adult CNS is highly controversial. For example, Hall and colleagues published that pericytes are critically involved in the regulation of CBF [16] which was also supported by Kisler et al. [17]. However, Hill et al. [18] found that arteriolar smooth muscle cells; and not pericytes, regulate regional blood flow. Furthermore, recent evidence has suggested that there may be several types of pericytes with differing roles such as pre-capillary pericytes with smooth muscle actin (SMA), and capillary pericytes without SMA expression [19–22]. Some evidence suggests that astrocytes may regulate pericyte tone and therefore vascular tone [22]. Although pericytes may be involved in capillary alterations, it is true that the arteries and arterioles, which are covered with VSMCs, need to be regulated to bring about a substantial change to CBF. It is evident from contradictions in the literature that regulation of CBF is not a simple mechanism (or as illustrated in Fig. 2); rather it is a complex interplay between various cell types and signalling pathways with many gaps still remaining in our understanding of the exact mechanisms behind neurovascular coupling in health and disease.

### Neuroimaging techniques to study neurovascular coupling

In order to study and measure neurovascular coupling as well as structural alterations to the NVU in vivo, live neuroimaging techniques are used. Neuroimaging techniques allow (largely) non-invasive visualisation of the brain, and are typically employed clinically to aid the diagnosis of disease, as well as in research to understand brain function physiologically and pathologically. Many of the clinical techniques can also be effectively used in pre-clinical imaging of animal models in vivo in studies of neurovascular coupling and cerebrovascular pathologies.

Functional magnetic resonance imaging (fMRI) has been the neuroimaging technique of choice for studying brain function in humans and to some extent in rodent models [23]. Briefly, fMRI is based on nuclear magnetic resonance related to proton alignment with a magnetic field [24]. The most commonly used detection paradigm is that of blood oxygen level dependent (BOLD) contrast. BOLD exploits the magnetic differences between oxyhaemoglobin (HbO) and deoxyhaemoglobin (HbR). In a resting state, the relative ratio between HbO:HbR is equal, therefore the magnetic field is unaffected [25]. Upon neural activation and consequent increased CBF, the proportion of HbO increases relative to HbR therefore creating an inhomogeneity in the magnetic field [25], which can be detected by a fMRI scanner. A positive BOLD signal generally reflects increased neural activation, whereas a negative BOLD signal may reflect decreased neural activity, and therefore blood flow [26], although this relationship is not always truly reflective of underlying neural activity especially in cases of pathology or specific cell type activation. fMRI can be used in both human subjects for psychiatric/psychological studies of brain function, clinically in the diagnosis of neurological disease, as well as pre-clinically in animal models.

fMRI and related BOLD-based imaging techniques make inferences of neural activity by measuring corresponding haemodynamic changes and do not measure neural activity directly. Neural activity can be recorded by directly measuring action potentials (spiking activity), or the summation of electrical activity determined by local field potentials (LFPs). In order to measure the electrical activity of neurons in vivo, multichannel microelectrodes are implanted in the brain region of interest, or on top of the scalp, producing electrical waveform data [27]. Correlating electrical neural activity to haemodynamic data enables direct association of neural events to corresponding haemodynamics in the study of neurovascular coupling, and is important to do so as blood flow may or may not be directly related to changes in neural activity.

To overcome some of the limitations of individual techniques and to provide a comprehensive representation with respect to brain activity, a multimodal approach is typically used in the study of neurovascular coupling with the combination of two or more techniques. For example, 2D-OIS (optical imaging spectroscopy) or BOLD-fMRI can be combined simultaneously with a micro-electrode implant for electrophysiology, or surface electroencephalography (EEG) into give both a haemodynamic and neural response [26, 28]. For example, a recent study has shown that multiple sclerosis (MS) patients show reductions in gamma power (through magnetoencephalography; MEG) as well as a reduction in BOLD (by fMRI) and CBF responses to visual stimulation [29]. It is important to consider not only a BOLD (blood oxygen level dependent)-related haemodynamic response in neuroimaging, but the source of neural activity itself, as blood flow changes often lag with respect to neural activity and may even decouple substantially in neurological disorders such as Alzheimer’s disease (AD) [17, 30].

At rest, neurons typically have a low basal [Ca^2+^] concentration of appropriately 50 nM, which is kept constant due to the action of several calcium ion channels and pumps such as the efflux plasma membrane calcium ATP-ase (PMCA) [31]. Upon neural activation (propagation of an action potential), influx calcium channels such as voltage gated calcium channels (VGCCs) and glutamate-mediated NMDA receptors, allow a significant influx of intracellular calcium with levels rising to 100-fold higher than baseline [32]. Therefore, neural activity directly correlates with changes to [Ca^2+^] levels. These indicators can be virally injected into the brain, or genetically engineered. Genetically encoded calcium indicators (GECIs) are bioluminescent protein complexes with integral fluorophores, which when stimulated emit a resonance signal in the form of visible light which can be detected by a camera, allowing quantification of neural activity based on the level of luminescence emitted by the fluorophore. Examples of common GECIs include *Yellow Cameleon 3.60* (YC3.60), which contains an enhanced cyan fluorescent protein (ECFP) donor fluorophore, bound to a Venus protein acceptor fluorophore, linked by a calcium-binding domain, calmodulin [33]. More recent GECIs include modifications to the GCaMP family, such as GCaMP6, which is a class of single-fluorophore GECI, where calcium binding causes a conformational change in the integral fluorophore (EGFP) to increase its own intensity [34]. Specific GECIs and other fluorescent proteins can be used to label specific and multiple cell types of the neurovascular unit simultaneously. This can also be achieved by 2D-OIS by measuring GCaMP-labelled neuronal activity, whilst simultaneously measuring haemoglobin reflectance changes, as done in Hillman’s group [35] amongst others.

2-photon microscopy is a technique in which a single laser emits two infrared (low-energy) photons to a focused region to cause excitation in fluorophores to a higher-energy state [36]. As the fluorophores return to their resting ground state they emit a light signal which is detected by a scanner. Calcium labelling of neurons and glial cells allows quantification of neural activity by measuring transient changes in spiking activity [32]. Fluorescent labelling of other cell types, including endothelium allows simultaneous blood flow measurements (RBC velocity), and therefore neurovascular coupling at the cellular level [37]. Multi-labelling in live animals eliminates the need to attach electrodes to the area of interest, therefore allowing neural and haemodynamic measurements to be taken by 2-photon microscopy over long periods of time. For example, it has been shown that amyloidosis (visualised by Methoxy-X04) around cerebral vasculature (visualised by TRITC-Dextran) disrupts the NVU function by a displacement of astrocytic-end-feet (visualised by Rhod-2 AM-loaded astrocytes,) by reducing overall CBF (determined by RBC velocity) in an AD mouse model [38]. Despite robust neural activity being present, the slow-down of CBF marks a decoupling of neurovascular coupling in AD.

Most stimulation paradigms in pre-clinical research involves the mechanical stimulation of either whiskers or fore/hind-paws to evoke a neural and haemodynamic response in the somatosensory cortex to study neurovascular coupling. Optogenetics involves expressing light-responding proteins such as channelrhodopsin 2 (ChR2), a blue-light sensitive protein which upon stimulation causes labelled cells to become active [39]. Neurons, as well as astrocytes, interneurons or even pericytes can be tagged with ChR2 in the cell-specific optogenetic stimulation approach. Adapting the correct filters in the 2D-OIS setup can allow the simultaneous optogenetic stimulation by blue light whilst also measuring haemodynamic changes as well as GECI-labelled neurons, in a shift to an all-optical approach to studying neurovascular coupling in vivo.

### Neurovascular dysfunction in neurological disorders

Many neurological conditions including the common neurodegenerative disorders display some level of neurovascular dysfunction; which either initiates or contributes to their pathogenesis, or is caused as a consequence. The neurovascular dysfunction seen in disorders such as Alzheimer’s and stroke can be directly attributed to cardiovascular deficits, however in some cases it may also not be directly associated with any underlying cardiovascular pathology, rather a functional breakdown of the neurovascular unit and signalling pathways.

#### Dementia (Alzheimer’s disease)

For the first time since medical records began, dementia has become the single-most leading cause of death in the UK surpassing heart disease, strokes and major cancers [40]. With an ever-ageing population, higher life expectancy and advances in diagnosis techniques, dementia currently affects as many as 1 in 6 in the UK [40]. Of the dementias, AD is the most prevalent followed by vascular dementia, whereas the remainder of cases comprise of frontotemporal dementia (FTD), dementia with Lewy bodies (DLBs) and early-onset dementia. In most of these cases, a mild cognitive impairment stage usually occurs much earlier on before the onset of dementia.

**Fig. 3:**
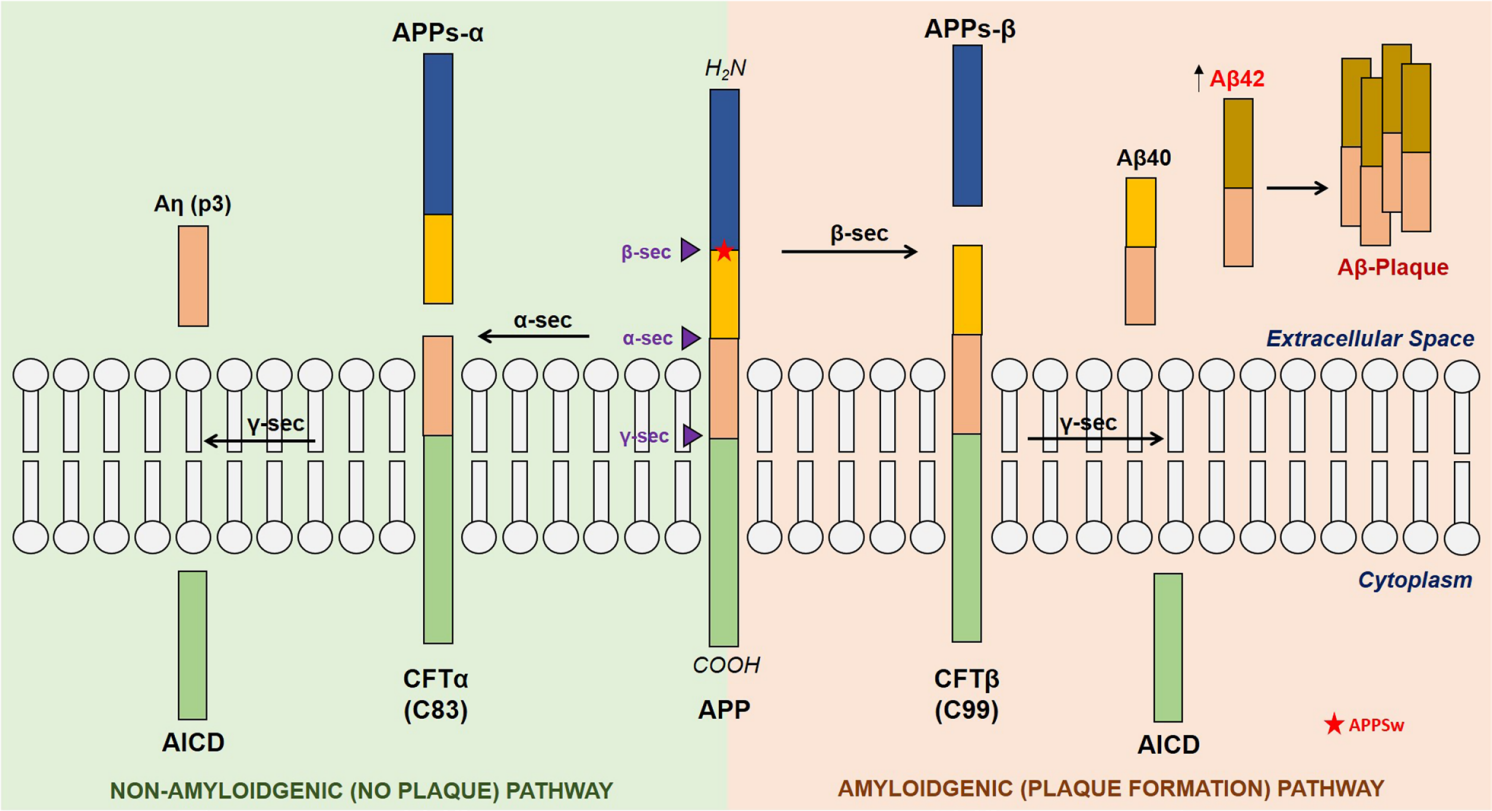
Overview of Aβ processing. APP can be processed via the α-secretase pathways (non-amyloidgenic pathway) producing the soluble APPs-α fragment and a small p3 fragment. The amyloidgenic pathway utilises β-secretase to produce the soluble APPs-β fragment followed by γ-secretase producing Aβ40 or lower in a much higher ratio than the production of Aβ42. Mutations to PSEN1/2 and APP (such as the Swedish mutation, marked by a red star on APP) often favour the production of the more toxic Aβ42 fragment which forms oligomers leading to the formation of Aβ-plaques. Adapted from [51, 70]

AD is an age-related neurodegenerative disorder and the most frequent form of dementia worldwide [41]. The primary pathological hallmarks of AD consist of extracellular β-amyloid (Aβ) plaques and intracellular neurofibrillary tangles (NFTs) of hyper-phosphorylated tau [42]. Familial cases of AD arise in an autosomal dominant inheritance pattern due to mutations to either amyloid precursor protein (APP), presenilin 1 (PSEN1) and presenilin 2 (PSEN2) [43–45]. Familial AD is usually relatively early-onset, however only accounts for around 10% of all AD cases worldwide [46]. Although genetic mutations in AD account for a small proportion of all AD cases, most of our understanding of AD pathology and mechanisms have come from animal models with these genetic mutations, for example the J20 mouse (PDGFB-hAPPSwInd) harbours the more common APP mutation called the *Swedish K670* *N/M671L* mutation as well as the *Indiana V717F* mutation, which both increase β-secretase activity [47, 48]. The increased production of the toxic form of amyloid, Aβ42, as well as Aβ40, coupled with the decreased ability to clear aggregates is the primary cause of Aβ-plaque build-up in the pathogenesis of AD [49]. Figure 3 illustrates the amyloidgenic processing pathway in the formation of Aβ plaques.

The vast majority of AD cases are sporadic (~ 90%) and are relatively later-onset, and are therefore not attributable to inherited genetic mutations to amyloid processing genes. It is important to note that even in sporadic AD cases, β-amyloid plaques are still present, and has many similarities to familial AD. Amongst the common risk factors already associated with cardiovascular disease and dementia such as age, hypertension, hypercholesterolaemia, diabetes etc., there are several risk factor gene polymorphisms which greatly enhance the likelihood of developing AD. The most common is the APOEε4 allele which is attributed to up to approximately 50-60% of all AD cases [50] as carrying the allele (found in around 15% of the population) increases the risk of developing AD fourfold, and homozygous inheritance of the APOEε4 increases the risk ninefold [50]. APOEε4 AD-patients have amyloid-β plaques as well as phosphorylated-tau NFTs similar to familial AD [51, 52]. The knowledge gained from the use of genetic models of AD therefore also applies to a vast proportion of all AD cases where amyloid is present.

There is a growing body of evidence which suggests that neurovascular coupling may be impaired in AD [21, 30, 53, 54]. It is postulated that impaired blood flow responses to neural activity as a result of neurovascular disarray may cause a mismatch between neural activity and provision of oxygen and glucose to meet sufficiently adequate metabolic demands. Consequentially, neural activity is reduced and thus correlates with impaired cerebral function, manifested clinically as cognitive decline [30]. As discussed previously, pericytes are critical in the integrity and maintenance of the BBB and therefore the NVU. Recent evidence has shown that patients with AD carrying the APOEε4-allele have a BBB breakdown attributed to pericyte degeneration in which patients/carriers have up to 50% less pericyte coverage correlating with almost 300-400 mm/mm^3^ total capillary length reduction compared to control subjects without the APOEε4 allele [55]. This study is further supported by Kisler et al. [17] in which they found that PDGFRB^−/−^ (pericyte deficient) mice have a reduced cerebral oxygenation level compared to controls as well as neurovascular uncoupling leading to neuronal death [17]. More recent evidence has directly implicated pericyte degeneration in extensive white matter pathology characterised by hypoxia and loss of myelination, leading to the loss of structural and functional connections within the brain which are typically present in some AD cases [56]. The breakdown of the NVU (pericytes amongst other cell types within the NVU) and the BBB is evident in Alzheimer’s patients, and may contribute to exacerbated pathology seen in AD, as well as initiating neuropathology. This can be further explained in the 2-hit vascular hypothesis model of AD proposed by Zlokovic [57] (Fig. 5). The first ‘hit’ refers to vascular dysfunction caused by various risk factors including atherosclerosis leading to a breakdown of vascular integrity causing reduced CBF which in itself can cause stroke (discussed below), cognitive decline and vascular dementia. The second ‘hit’ refers to the increased Aβ levels (by a reduction in clearance) which further exacerbates neuronal dysfunction and adding further insult to the pathogenesis of AD [57]. It is therefore becoming increasingly more apparent that AD is not solely a neurodegenerative disorder, rather it is a complex multifactorial disease with a large neurovascular element which needs to be investigated further both in disease initiation and in potential therapeutic strategies.

#### Stroke

Vascular dementia and other forms of cognitive impairment can be caused directly by having a stroke. Strokes can arise as a direct consequence of arterial pathology with profound changes to the neurovascular unit and the normal physiology of neurovascular coupling. This impaired blood flow to the brain can lead to the death of neural tissue in specific regions where blood flow is disrupted to cause a stroke. Depending on the severity of the stroke, symptoms can be mild ranging from a transient ischaemic attack to a potentially fatal in a stroke where a large are of the brain is affected [58]. Strokes can fall into two broad categories: ischaemic, where occlusion of vessels leads to death of tissue; or haemorrhagic as a result of a ruptured vessel [58]. As a consequence of stroke, cognitive and motor function can become impaired, sometimes permanently leading to vascular dementia for example.

Subarachnoid haemorrhages (SAH) as a result of cerebral aneurysm rupture can cause a devastating stroke which usually results in death a few days after the initial rupture, if not fatal during the initial stroke [59]. It has been demonstrated that in such haemorrhagic episodes, there tends to be an inversion of neurovascular coupling in response to calcium-signalling mediated by astrocytes [59]. Instead of Ca^2+^-mediated vasodilation, post-stroke, there is a switch in the polarity of neurovascular coupling in which Ca^2+^ actually causes vasoconstriction pathologically. This then limits CBF and functional hyperaemia to the brain parenchyma resulting in progressive ischaemia after the stroke takes place usually resulting in death of the patient a few days after the stroke [59]. A potential mechanism as to why this may occur may be due to the upregulation of astrocytic endothelin-1 (ET-1); a vasoconstrictor, after SAH [60]. ET-1 binds to ET_B1_ receptors on endothelial cells to reduce overall synthesis of endothelial nitric oxide synthase (eNOS), and hence NO levels. ET-1 also binds to ET_A_ receptors on the tunica media of vessels to upregulate PKC-α, which in turn inhibits the function of K^+^ channels to contribute to vasospasm, and BBB breakdown causing oedema [60]. These studies highlight the importance of astrocytes in neurovascular coupling by alluding to the critical signalling pathways between neurons-astrocytes-endothelial cells in facilitating vasodilation, as well as providing insight into the complexity of stroke pathophysiology.

#### Physiological ageing

Ageing is itself the biggest risk factor in the development of many diseases including cardiovascular disease, Alzheimer’s and vascular dementia. Normal physiological ageing itself results in progressive neurovascular dysfunction, primarily marked by a reduction of overall CBF [61, 62]. A key reason why many age-related disorders begin is due to the accumulation of reactive-oxygen species (ROS) coupled with impaired ROS-scavenging. Indeed, it has been shown that an enhanced production of ROS leads to an overall reduction in NO within the vasculature, therefore impacting NO signalling to cause vasoconstriction over time [10]. This arises as a direct result of aged vessels affecting their structure and function.

A recent study by Duncombe and colleagues explored the pathophysiology of neurovascular coupling associated with ageing [63]. They found that aged mice show key differences in the NVU compared to younger mice. Firstly, pericyte coverage is markedly reduced in older mice [63]. However, this study did not find any correlative effects of the loss of pericytes with CBF and alterations to neurovascular coupling. This is contradicted by another study by Kisler and colleagues [17], which found that the loss of pericytes directly causes neurovascular uncoupling, in addition to reduced oxygen perfusion in the brain [21]. As alluded to earlier, studying the role of pericytes in neurovascular coupling is a challenging task, mainly as there are few specific markers for pericytes, as well as limited scope to study blood flow in capillaries in vivo, which is acknowledged by Duncombe. Secondly, Duncombe and colleagues found that aged mice exhibit astrocyte end-feet abnormalities by the marked reduction in the expression of AQP4 [63], which impairs the cell–cell communication between astrocytes and endothelial cells via the neurogliovascular signalling pathways (as shown in Fig. 2). Ageing, therefore, progressively impairs functional hyperaemia by a wide variety of pathophysiological changes associated with the NVU, in addition to the loss of key signalling pathways, which may be further worsened in AD through excitotoxic calcium signalling due to Aβ plaque pathology [64].

### Atherosclerosis

One key pathological change associated with ageing is that of atherosclerosis. Atherosclerosis is a chronic inflammatory syndrome resulting in the progressive thickening and hardening (arteriosclerosis) of major artery walls [65]. This continual narrowing of the lumen restricts blood flow over time, and is causative in millions of deaths worldwide each year with the commonest causes of death being stroke, myocardial infarction and dementia [66]. The aetiology of atherosclerosis is complex with a combination of genetic, lifestyle (e.g. dietary), environmental and immunological factors contributing towards its pathogenesis and progression.

Atherosclerosis can take decades to take full effect with patients being largely asymptomatic until their mid-50 s, although initiation of disease takes place in early adulthood. The initiation of atherosclerosis is still not fully understood, although general consensus is that atherosclerosis begins with inflammatory changes within the endothelium caused by low-density lipoproteins (LDLs) being deposited [67, 68], and internalised by endothelial cells. However, what is becoming increasingly clearer is that deposition of LDLs into the arterial walls is not sufficient to cause atherogenesis, rather it is the subsequent immune response facilitated by monocytes and leukocytes that initiates the syndrome [69], Fig. 3.

LDLs have been shown to deposit more readily in atherosclerotic-prone and low blood flow areas of an arterial branch, where they undergo oxidation by reactive oxygen species (ROS), stimulating an immune-response [70]. The arterial branches susceptible to LDL deposition and atherosclerosis tend to be near areas of major curvatures (e.g. carotid sinus) which have a high shear index compared to laminar areas of the same arterial tree, that tend to be more athero-reistant [71].

**Fig. 4:**
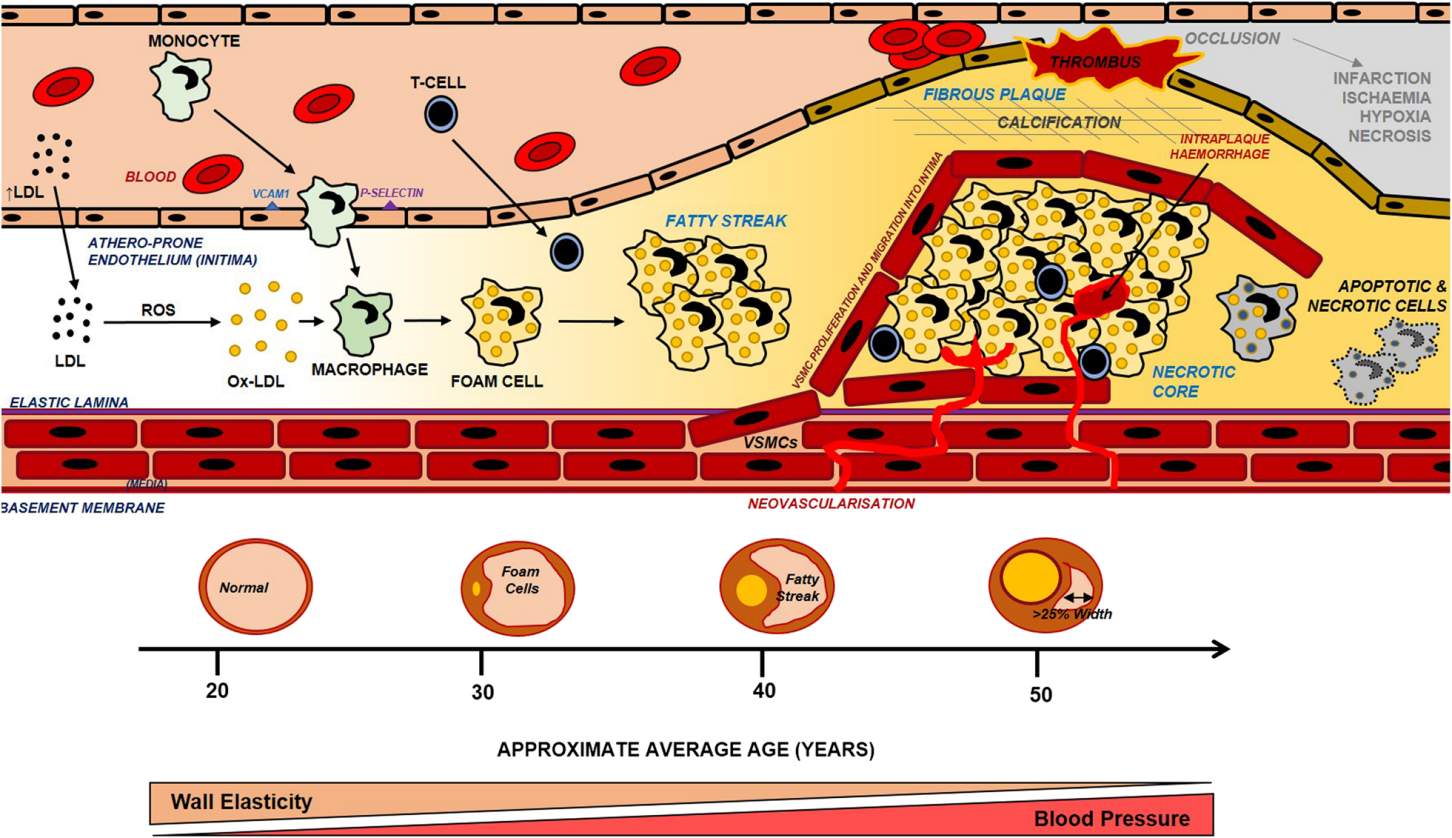
Overview of the pathogenesis of atherosclerosis. Accumulation of LDLs in the blood leads to LDLs being transported into the endothelium, where they become oxidised. Infiltrating monocytes become activated and differentiate into macrophages and begin to ingest oxidised-LDLs to become foam cells. Over time, foam cells conglomerate to form fatty streaks. Simultaneously, due to inflammation and stimulant factors, VSMCs begin to proliferate and migrate into the intima and form a cap around the fatty streak. Endothelial cells overlying the growing plaque become calcified, with reduced elasticity. Over time this cap restricts blood flow due to a luminal constriction. If the plaque ruptures, a thrombus forms and obstructs the lumen completely, or partially. This can lead to ischaemia downstream leading to an ischaemic attack in the brain, or rupture to cause a haemorrhagic stroke. Adapted from [107]

The solidifying step in atherogenesis is when invading monocytes differentiate into macrophages in the intima after binding to endothelial receptors such as VCAM1 and P-Selectin [72], Fig. 4. Once differentiated into macrophages, oxidised-LDLs are subsequently engulfed, slowly transforming normal macrophages into ‘foam-cells’ with a large accumulation of lipids intracellularly [73]. Over time, clusters of foam cells form visible ‘fatty-streaks’ inside the intima of the endothelium. Simultaneous to fatty-streak formation, endothelial VSMCs also begin to proliferate and migrate from the media into the intima and form a ring around the fatty-streaks further stabilising the forming plaque [74]. Over time some of the VSMCs begin to calcify and harden adjacent to the atheromatous endothelium, forming a solid core in the intima of the endothelium, which begins to enlarge and partially occlude the vessel to cause a progressive narrowing (stenosis) of the artery [75].

The atheromatous plaque (atheroma) may in itself cause an obstruction of blood flow, or more commonly the atheroma may rupture and form a thrombus with platelets, which can be repeated to form a stable cap disrupting blood flow over time further exacerbating stenosis, or rupturing within the vessel wall to cause either an embolism or haemorrhage. Either of these can contribute to coronary heart disease, myocardial infarction (if in the heart), or a stroke if in the carotid or cerebral arteries.

### Atherosclerosis and neurovascular dysfunction

Atherosclerosis affects most large to medium sized systemic arteries including those supplying the cerebral circulation, namely the internal carotid and vertebral arteries [58]. Progressive plaque build-up and stenosis of cranial arteries can cause strokes as well as contribute to the development of dementia either directly or as a consequence of stroke. Strokes can either be ischaemic or haemorrhagic in nature, and can both trace their origins to underlying vascular pathologies, such as luminal stenosis as a consequence of atherosclerosis in the arteries supplying the brain and cranium [58]. Here, only the relationship between cardiovascular disease and cognitive impairment will be discussed.

**Fig. 5:**
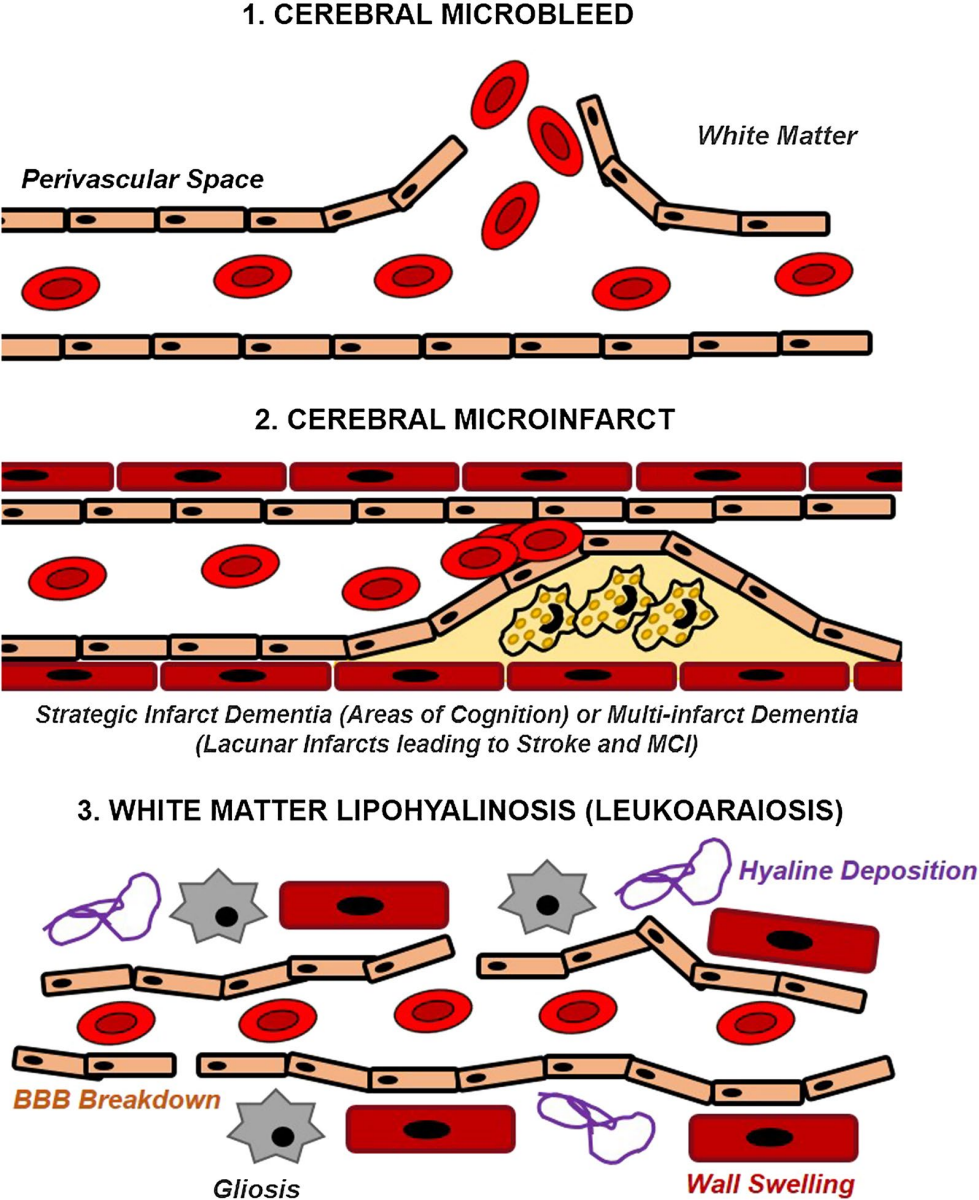
Vascular lesions in small vessel disease. Key types of vascular lesions found in the brain as a result of small vessel disease (SVD) leading to cognitive impairment (vascular dementia) and stroke include microbleeds, microinfarcts; where fatty deposits accumulate to restrict luminal diameter, and inflammatory white matter pathology such as lipohyalinosis, where hyaline deposits within connective tissue in addition to astrocytic gliosis (scarring) and wall swelling to disrupt the uniformity of vessel walls within the brain. Adapted from [76]

Atherosclerosis and related cardiovascular diseases and risk factors can cause a wide array of simple and complex vascular lesions in the brain. For example, bilateral carotid occlusion directly affects the blood supply to the brain, and is commonly associated with stroke and vascular dementia [76]. Some of the key vascular lesions commonly found in patients with cardiovascular pathology range from microbleeds, microinfarcts (lacunar multi-infarct or strategic) and lipohyalinosis; the deposition of hyaline in the connective tissue walls to disrupt the integrity of the vasculature, affecting the smaller vessels within the white matter termed cerebral small vessel disease [77], refer to Fig. 5. These lesions arise directly as a consequence of arterial stiffness and inflammation caused by atherosclerosis and vascular risk factors including hypertension, hypercholesterolaemia, smoking, diet and advanced age [78]. Cerebral small vessel disease is directly associated with the onset of dementia, primarily vascular dementia as well as Alzheimer’s-like pathology and other milder forms of cognitive impairment as well as stroke. Depending on where in the brain these vascular lesions occur, different symptoms may manifest clinically. Pre-symptomatic (subclinical) carotid atherosclerosis (determined by increased endothelial swelling Fig. 5) is associated with early cognitive impairment, in which pathologic mechanisms have been attributed to cerebral microvascular dysfunction (as shown in Fig. 5) in the frontal and temporal lobes [79].

Lipohyalinosis is a brain-specific vessel disease characterised by vascular wall disruption and occurs as a result of systemic atherosclerosis, especially due to extensive inflammation occurring through the accumulation of ROS, in addition to gliosis involving astrocytes, oligodendrocyte precursor cells and microglia which themselves in their reactive state produce ROS [80]. As discussed previously, atherosclerosis is an inflammatory syndrome which initiates as a result of an immune response against oxidised-LDLs in the intima. Monocyte infiltration into the vessel wall followed by differentiation into macrophages which subsequently engulf the oxidised-LDLs forming foam cells is the primary inflammatory trigger [69]. In addition to monocyte infiltration at sites of LDL retention, T_H_1 (CD4^+^) lymphocytes are also involved in the inflammatory response [72, 81]. T_H_1 cells bind to antigen sites on LDL molecules and subsequently produce inflammatory mediators including tumour necrosis factor (TNF) and interferons e.g. IFNγ [72], which all promote the pathogenesis and development of atherosclerosis. Cholesterol crystals activate the NOD-like receptor protein 3 (NLRP3), which together with caspase-1 and apoptosis-associated speck-like protein containing a caspase activation and recruitment domain (CARD) forms an inflammasome [82]. This stimulates the production of the proinflammatory IL-1β chemokine. IL-1β has been shown to be implicated in the pathogenesis of atherosclerosis and thrombosis by the downstream stimulation of IL-6 and C-reactive protein (CRP) [83]. Recent evidence has suggested that targeting the inflammatory pathways in atherosclerosis irrespective of reducing LDL-C levels, may actually be therapeutically beneficial in patients with atherosclerosis, by reducing the frequency and strength of major cardiovascular incidents [83, 84]. Clinical trials involving canakinumab, a monoclonal antibody against IL-1β, has shown a significant reduction of major cardiovascular incidents in patients with atherosclerosis, by reducing the total levels of plasma IL-6 and CRP compared to those given a placebo [83].

**Fig. 6:**
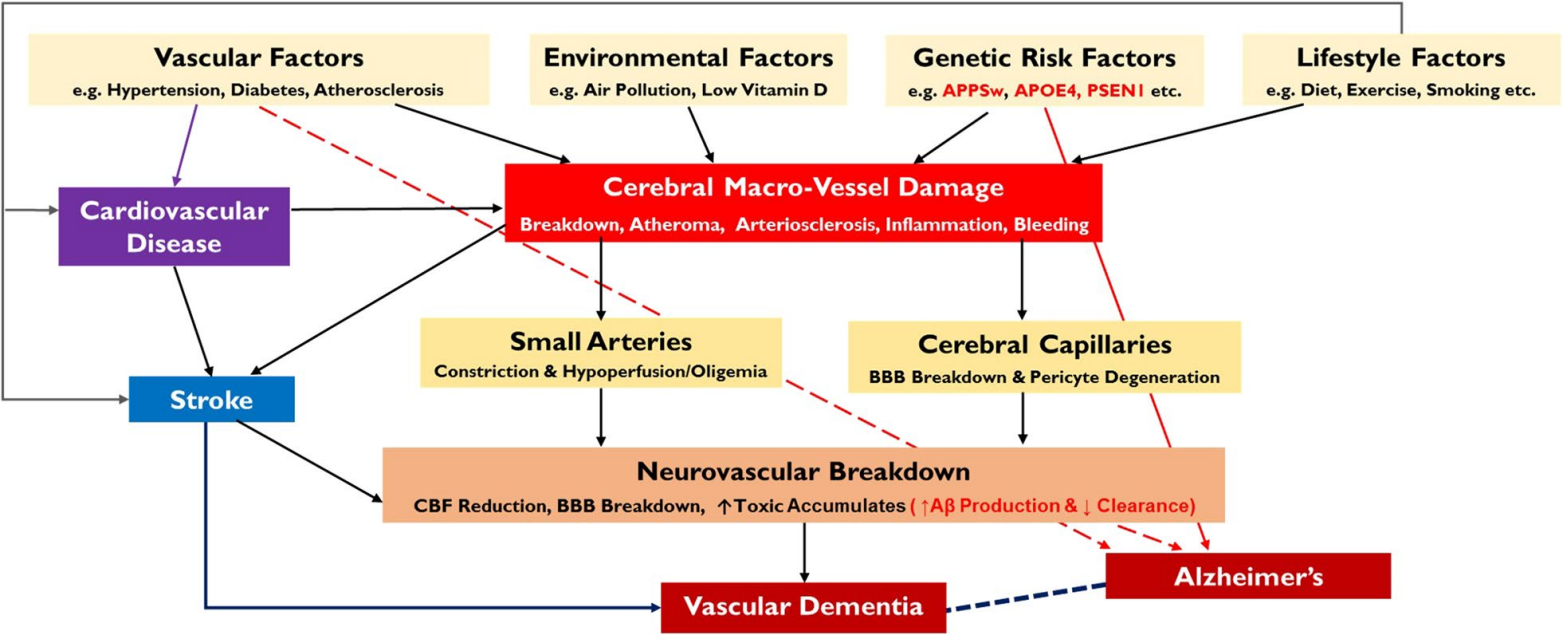
Summary of vascular pathologies in CVD, stroke and dementia. Vascular dysfunction pathway for stroke, vascular dementia and AD; AD-specific factors in red text (incorporating the 2-hit hypothesis for AD—hit 1 being at vessel damage and hit-2 being Aβ increase). CVD can directly cause cerebral vessel damage to cause either strokes and/or vascular dementia, in addition to initiating AD pathology if certain risk factors are present. Adapted from [21, 108]

Vascular dysfunction caused by atherosclerosis and lifestyle/genetic risk factors can directly lead to cerebral vascular damage to cause small vessel disease and a breakdown of the BBB, marked by inflammation and hyper-connectivity [21]. This vascular damage leads to neurovascular dysfunction marked by CBF decrease (oligemia), as well as potentially initiating Aβ pathology by a reduction in clearance. Figure 6 illustrates how AD can develop (including the 2-hit vascular model proposed by Zlokovic [57]) as a result of vascular dysfunction leading to amyloid production, in addition to the pathogenesis of vascular dementia at the level of neurovascular dysfunction, independent of amyloid pathology. Figure 6 also summarises the key processes involved in atherogenesis and vascular dysfunction, including inflammation, showing the complex overlap between disease conditions.

### LDLR, ApoE and PCSK9

LDL molecules comprise of an apolipoprotein B-100 protein, in addition to other lipids (including up to 1500 cholesterol molecules), triglycerides and structural protein complexes [85]. LDLs bind to the LDL-receptors (LDLRs) in hepatocytes, primarily, via the ApoB segment to stimulate endocytosis of the LDLR/LDL complex [86]. The closely related ApoE protein, which also binds to LDLR, is involved in the selective transport of lipoproteins systemically, especially in the transport of chylomicron and LDL remnants [87]. Genetic mutations that cause a loss of function to either the LDLR, ApoE or ApoB of an LDL molecule result in the reduced clearance of LDL from the plasma, therefore increasing the circulating plasma levels of cholesterol [88]. Whilst mutations to these genes may cause familial hypercholesterolemia from a much earlier age, affecting as many people as around 1:500 (heterozygotic) [88], non-affected individuals can naturally develop hypercholesterolemia and therefore atherosclerosis with elevated LDL levels either through dietary intake of trans and saturated fats, being obese, in addition to having diabetes, or simply being male [88]. ApoE^−/−^ and LDLR^−/−^ knockout mice can develop hypercholesterolemia and atherosclerosis and disease severity and progression is exacerbated by being fed a high-fat diet [89, 90]. ApoE^−/−^ mice also develop BBB leakage which worsens over time, and may contribute to a breakdown of neurovascular coupling [91, 92].

**Fig. 7:**
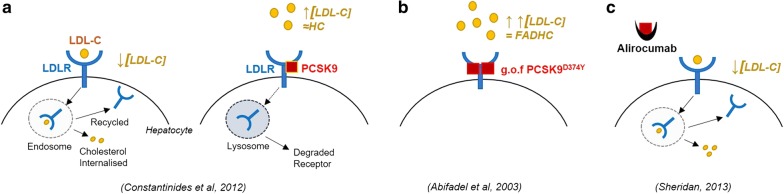
PCSK9 mechanisms. *LDL* low-density lipoprotein, *LDL*-*C* cholesterol, *LDLR* low-density lipoprotein receptor, *PCSK9* proprotein convertase subtilisin/kexin type 9, *HC* hypercholesterolemia, *g.o.f*. gain of function, *FADHC* familial autosomal dominant hypercholesterolemia. **a** (Left)—physiological homeostatic regulation of LDL-C by LDLR. **a** (Right)—physiological homeostatic regulation of LDLR by PCSK9 (which may lead to HC). **b** Genetic mutant (g.o.f.) variant of PCSK9 causing FADHC. **c** PCSK9 inhibitor mechanism to reduce LDL-C levels in patients with HC

More recently, an rAAV8 D377Y-mPCSK9 (PCSK9^DY^) mouse model (on a male C57BL/6 background) has been used to study atherosclerosis [93–95], where a single injection of AAV-mPCSK9^DY^ is sufficient to cause long-term atherosclerosis in mice comparable to LDLR^−/−^ strains, without the need to create a transgenic mutant knock-in line [93]. The proprotein convertase subtilisin/kexin type 9 (PCSK9) gene on chromosome 1 (1p32.3) has been shown to homeostatically regulate cholesterol levels in the plasma by directly interacting with the LDL-receptor (LDLR) and the related APOE-receptor (ApoER_2_) by causing their internalisation and degradation within a cell [96, 97]. Over-time, the degradation of LDLR with respective increased plasma cholesterol levels may be causative to atherosclerosis in ordinary individuals [98]. Gain of function mutations to PCSK9 (e.g. D374Y) are associated with patients that have genetic hypercholesterolemia, in particular, familial autosomal dominant hypercholesterolemia (FADHC) [99]. In the case of the human PCSK9^D374Y^ mutation, plasma concentrations of cholesterol can exceed 500 mg/dL, where normal levels would be less than 200 mg/dL and pathological being above 250 mg/dL [99]. Recently it has been shown that there is significantly increased PCSK9 within the CSF of Alzheimer’s patients carrying the APOEε4 allele, with a yet unknown pathway [100], further adding support to the notion that hypercholesterolaemia is a major risk factor for AD. With the knowledge that PCSK9 is involved in elevated cholesterol levels both physiologically and pathologically, monoclonal antibodies and selective inhibitors, such as alirocumab (against PCSK9), have been approved by the FDA for the treatment of patients with hypercholesterolemia and atherosclerosis by halting the progression of cardiovascular disease [101]. Figure 7 illustrates the physiological and pathological mechanisms of PCSK9 in the regulation of plasma cholesterol levels. It has been shown that a high-fat diet exacerbates atherogenesis by elevating total cholesterol levels in PCSK9^DY^ mice, much like the LDLR^−/−^ strains, however normal rodent chow can also result in moderate atherogenesis [95]. As this is a relatively novel atherosclerosis model, not much is known about the effect on the brain or neurovascular coupling. This viral injection method of inducing atherosclerosis in any strain of mouse is far cheaper than breeding and maintaining inbred colonies, and it is becoming the model of choice due to its rapid induction (including in any genetic-model), reliable and consistent atherosclerotic profiles as well as cost. Compared to the artificially high serum cholesterol levels seen in the ApoE^−/−^ mice, and having the added benefit of not maintaining knockout lines, the rAAV-PCSK9^DY^ model is certainly the more economical and more effective model in terms of induction of atherosclerosis as well as a comparable LDL-C profile to that of patients.

It is important to note that all animal models of human disease can never completely replicate human disease in vivo, due to species-specific physiologies; therefore, the models that we do have are our best insight into a more complex scenario with respect to human disease, and results must be interpreted with some level of caution before generalising both in terms of knowledge gained and potential for therapeutics. Although animal models can model human disease and provide insights into disease mechanisms effectively, sometimes the same model may provide conflicting evidence in the hands of other researchers. Studies have found a link between hypercholesterolemia and the onset of vascular dysfunction causing Alzheimer’s(-like) pathology in patients [102], as well as in high-fat diet mouse models [103]. However, in a study by Hohsfield and colleagues [104], it was shown that hypercholesterolemia does not increase amyloid-deposition and Alzheimer’s like pathology in the same 3xTg-AD mouse model. However, unlike in the previous studies, the 3xTg-AD mice in Hohsfield’s study did not survive long enough, therefore probably did not gain enough cumulative amyloid/tau deposition to manifest as Alzheimer’s-like pathology. They did not consider more regular behavioural assays such as Morris maze tests to establish whether their mice displayed any early mild cognitive deficits.

## Conclusion

Neurovascular coupling is critical to the proper functioning of the brain, ensuring that increased neural activity, which itself is incredibly metabolically demanding, is matched with an appropriate change to CBF to both nourish the parenchyma with oxygen and glucose as well as the quick and efficient removal of carbon dioxide and waste products. In order to achieve this, neurons communicate with endothelial cells via the neurogliovascular signalling pathways within the NVU. Pathological changes to the NVU and endothelia impairs the signalling pathways involved in neurovascular coupling, leading to brain pathologies ranging from subtle cognitive deficits to severe AD. Although our knowledge and understanding of the mechanisms behind neurovascular coupling in health, ageing and disease has vastly improved in recent years, much is still to be investigated, especially with respect to the effect of atherosclerosis on cerebral vasculature and neurovascular coupling. Although there are many established models of atherosclerosis, the new rAAV-PCSK9^DY^ method has many benefits over the conventional knock-out lines of ApoE^−/−^ and LDLR^−/−^, including the ability to induce atherosclerosis in any mouse model including AD-mouse models. In future studies, tracking haemodynamic changes over time combined with cellular, genetic and in vivo imaging would provide a comprehensive overview and mechanistic insight into how cardiovascular disease affects neurovascular coupling and the brain as a whole in mouse models of atherosclerosis and mixed models of dementia.
